# A novel role of centrin in flagellar motility: stabilizing an inner-arm dynein motor in the flagellar axoneme

**DOI:** 10.15698/mic2014.08.161

**Published:** 2014-07-14

**Authors:** Ziyin Li

**Affiliations:** 1 Department of Microbiology and Molecular Genetics, University of Texas Medical School, Houston, TX 77030, U.S.A.

**Keywords:** Centrin, inner-arm dynein, flagellar motility, axoneme, Trypanosoma brucei

## Abstract

Centrin is an evolutionarily conserved EF-hand calcium-binding protein found in the centriole of animals and the basal body of flagellated organisms. It was originally discovered in the flagellated unicellular green alga *Chlamydomonas* *reinhardtii*, where it associates with flagellum-associated structures and regulates basal body duplication and flagellar motility. Centrin constitutes a light chain of three inner-arm dynein complexes in the flagellar axoneme in *Chlamydomonas*, and presumably regulates the activity of the inner-arm dynein for flagellar motility. In the ciliated organism *Tetrahymena*, centrin also associates with the inner-arm dynein and appears to regulate the microtubule sliding velocity of the inner-arm dynein. Using *Trypanosoma brucei* as the model organism, we discovered that centrin maintains the stability of an inner-arm dynein in the flagellar axoneme [Wei *et al*., (2014) Nat. Commun 5: 4060]. *T. brucei* expresses five centrins, three of which, TbCentrin1, 2, and 4, associate with the flagellar basal body, but no centrin was found to regulate cell motility. We found that TbCentrin3 associates tightly with the flagellum and that RNAi of TbCentrin3 compromised cell motility. Biochemical approaches further showed that TbCentrin3 interacts with TbIAD5-1, an inner-arm dynein in the flagellar axoneme. Knockdown of TbIAD5-1 also caused defective cell motility. Strikingly, depletion of TbCentrin3 or depletion of TbIAD5-1 resulted in disassembly of the complex from the axoneme and subsequent degradation of the complex in the cytosol. Our findings identified a novel role of TbCentrin3 in cell motility by stabilizing TbIAD5-1 in the axoneme, which likely is well conserved in other flagellated and ciliated organisms, such as *Chlamydomonas *and *Tetrahymena *where centrin is also known to associate with inner-arm dyneins.

*Trypanosoma brucei* is an early branching unicellular eukaryote and the causative agent of sleeping sickness in human and nagana in cattle in sub-Sahara Africa. A trypanosome cell possesses a single motile flagellum that is nucleated by the basal body situated at the posterior portion of the cell and further extends to the anterior tip of the cell. The flagellum is composed of a canonical axoneme and an extra-axoneme structure termed paraflagellar rod (PFR) (Fig. 1A), in which intra-flagellum transport of cargoes is carried out. Like the axoneme of all the motile flagella in other flagellated organisms, the axoneme in trypanosome flagellum also consists of a ring of nine doublet microtubules surrounding a central pair of singlet microtubules. Outer dynein arm (ODA) and inner dynein arm (IDA) extend from each of the nine doublet microtubules and generate sliding forces that drive flagellar motility (Fig. 1A). The molecular composition of the outer dynein arms and inner dynein arms remains to be determined, but, based on the genomic data, *T. brucei* possesses two putative outer-arm dyneins and seven inner-arm dyneins. A number of putative dynein-associated intermediate chains, light chains and light-intermediate chains are also encoded by the *T. brucei* genome, but their respective dynein heavy chains are still not assigned.

**Figure 1:**
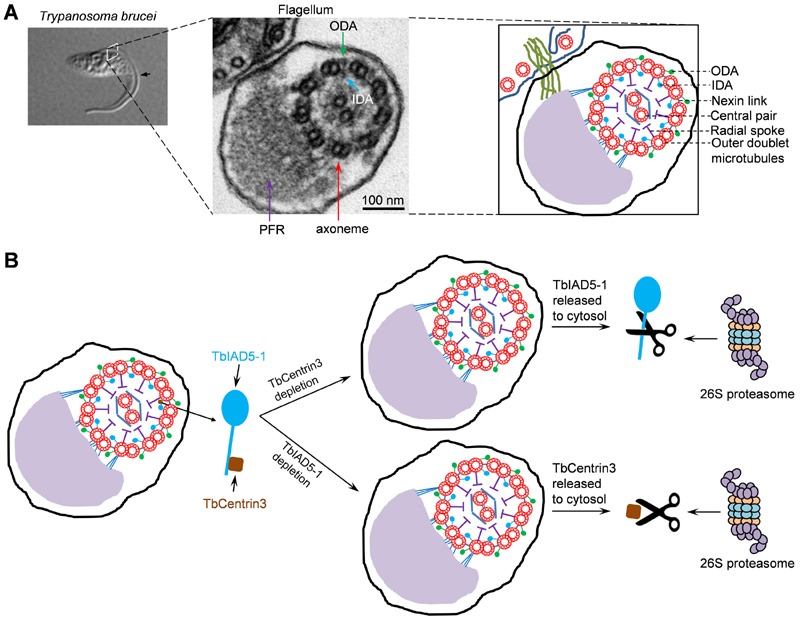
FIGURE 1: Role of TbCentrin3 in flagellar motility in trypanosomes. **(A)** The trypanosome flagellum and its axoneme structure. Shown on the left panel is a differential interference contrast (DIC) image of a procyclic cell of *T. brucei* with a single flagellum (arrow). The middle panel shows the transmission electron microscopy of a cross-section of the flagellum and the cell body to which the flagellum is attached. The paraflagellar rod (PFR), the axoneme, outer dynein arm (ODA), and inner dynein arm (IDA) are indicated. The right panel is a schematic representation of the micrograph shown in the middle panel. **(B)** Effect of TbCentrin3 depletion on the assembly and stability of TbIAD5-1 and vice versa. Note that only one IDA is labeled as the TbIAD5-1-TbCentrin3 complex and that TbCentrin3 and TbIAD5-1 are not drawn to the same scale. The precise numbers of inner dynein arms containing TbCentrin3 and TbIAD5-1 are still unclear. In the absence of TbCentrin3, newly synthesized TbIAD5-1 is not assembled to the axoneme and the old, axoneme-associated TbIAD5-1 is released to the cytosol, where it is degraded by the 26S proteasome. Conversely, in the absence of TbIAD5-1, new TbCentrin3 also fails to assemble to the axoneme and old TbCentrin3 is released from the axoneme to the cytosol, where it is degraded by the 26S proteasome.

The discovery of centrin in the inner-arm dynein complexes can be dated back to 1990. The single centrin protein in *Chlamydomonas reinhardtii* was found to associate with three distinct inner-arm dynein complexes. Similarly, centrin was also found in the inner-arm dynein complex in the cilium of *Tetrahymena*. Centrin in *Chlamydomonas* and *Tetrahymena* is also present in the basal body and is required for basal body duplication. In *T. brucei, *however, five centrin-like proteins are expressed. It is thus hypothesized that the five centrins may play specialized roles in *T. brucei*, which likely are executed by the single centrin in other unicellular organisms. Previous work demonstrated that three centrins, TbCentrin1, TbCentrin2, and TbCentrin4, associate with the flagellar basal body, and RNAi silencing of TbCentrin1 or TbCentrin2 inhibits basal body duplication. Intriguingly, TbCentrin2 and TbCentrin4 are also localized to a so-called bilobe structure, whose precise function is still not well understood. TbCentrin2 plays an additional role in controlling Golgi duplication, whereas TbCentrin4 appears to coordinate nuclear and kinetoplast segregation. Nothing is known about the localization and function of the two remaining centrins in *T. brucei*, TbCentrin3 and TbCentrin5, although previous proteomic studies suggest that TbCentrin3 associates with flagellum-related structures including basal body, flagellum attachment zone, and flagellum. Our research interest focuses on the cell cycle regulation in *T. brucei*, and tremendous efforts from several research laboratories have been made to delineate the signaling pathways driving the cell cycle progression.

However, although it is well accepted that basal body duplication/segregation constitutes one of the earliest cell cycle events in *T. brucei*, little is known about the coordination between basal body duplication/segregation and mitotic onset. We thus set out to characterize TbCentrin3 and, to our surprise, TbCentrin3 does not localize to the basal body, but instead is a flagellar protein involved in cell motility. TbCentrin3 was knocked down by RNAi, which was very effective, resulting in depletion of TbCentrin3 within 3 days of RNAi induction. Strikingly, while the TbCentrin3 RNAi cells apparently lost directional cell motility, they were still capable of dividing, albeit at a slightly reduced rate as compared to the non-induced control cells. This observation is consistent with previous findings that RNAi silencing of a number of flagellar proteins compromised cell motility but did not inhibit cell proliferation. Notably, despite the loss of cell motility, the flagella of TbCentrin3 RNAi cells still possess beating capability, but it is not clear whether the rate of flagellar beating was reduced or not. Nevertheless, the RNAi cells just tumble around or make circles or only travel a small distance. Electron microscopy showed that the "9+2" axoneme microtubules and the PFR structure were not altered. Moreover, there were no detectable defects in the outer dynein arms and inner dynein arms upon TbCentrin3 RNAi. Electron microscopy likely is not sufficient to detect any structural defects in the flagellum caused by TbCentrin3 deficiency.

In light of the association of centrin with the inner-arm dyneins in *Chlamydomonas* flagellum and *Tetrahymena* cilium, it is highly likely that TbCentrin3 also resides in the inner dynein arm(s). There are a total of seven putative inner-arm dyneins encoded by the *T. brucei* genome, but little is known about the molecular composition of these inner-arm dynein complexes and the function of these dyneins in cell motility. Presumably we could have tested which dynein(s) interacts with TbCentrin3 *in vivo* by co-immunoprecipitation. However, we set out to carry out tandem affinity purification to identify TbCentrin3-interacting protein(s) as this approach is straightforward and may identify non-dynein partner(s). Since TbCentrin3, and presumably its interacting partner(s), associates tightly with the flagellum and hence is detergent insoluble, we lysed the cells by thorough sonication through which the flagellar proteins are known to be released. A single high molecular mass protein was co-immunoprecipitated by TbCentrin3, which was further identified as one of the seven inner-arm dynein heavy chains, TbIAD5-1, by LC-MS/MS. Reciprocal tandem affinity purification with TbIAD5-1 as the bait also precipitated TbCentrin3, further confirming that the two proteins form a complex *in vivo* in *T. brucei*. These findings suggest a conserved involvement of centrin in the inner dynein arm in trypanosomes as in *Chlamydomonas* and *Tetrahymena*.

A few interesting findings were subsequently made when we examined the effect of TbCentrin3 RNAi on TbIAD5-1 localization and stability and vice versa. When TbCentrin3 was depleted from the cells, TbIAD5-1 was not assembled to the flagellar axoneme and, additionally, axoneme-associated TbIAD5-1 was released from the distal tip of both the new and old flagella, leading to accumulation of TbIAD5-1 in the cytosol where it was eventually degraded by the 26S proteasome (Fig. 1B). This unexpected finding suggests that TbCentrin3 maintains the stability of TbIAD5-1 in the axoneme and that the axonemal inner-arm dynein is assembled in a polarized fashion from the distal tip of the flagellum. Conversely, in the absence of TbIAD5-1, TbCentrin3 was also depleted from the flagellar axoneme in a uniform fashion without clear polarity. The mechanism underlying the distinction between TbIAD5-1 disassembly and TbCentrin3 disassembly is, unfortunately, still unclear. Nevertheless, like TbIAD5-1, TbCentrin3 was also released to the cytosol and was subsequently degraded by the 26S proteasome (Fig. 1B). These results provide strong evidence that the two proteins are mutually required for maintaining a stable inner-arm dynein complex in the axoneme.

The presence of centrin in the flagellar inner-arm dynein complexes has been known for almost 24 years. However, precisely how centrin regulates the inner-arm dynein and how centrin contributes to flagellar motility remain mysterious. Presumably, centrin may alter the microtubule sliding activity of the inner-arm dynein in response to the calcium, thus playing a regulatory role as a light chain of the inner-arm dynein complexes. Our findings identify an unexpected role of centrin in flagellar motility by stabilizing an inner-arm dynein. Such a role might be well conserved in other flagellated and ciliated eukaryotes as well, but due to the multiple roles played by the single centrin in these organisms, direct assessment of the effect of centrin deficiency in flagellar motility is not possible. However, because of the specialized roles of centrins in *T. brucei*, molecular dissection of centrin function in flagellar motility became feasible using *T. brucei* as the model system. Future efforts will be directed to characterize the contribution of TbCentrin3 to the microtubule sliding activity of TbIAD5-1 and to investigate the contribution of calcium binding to TbCentrin3 function in regulating TbIAD5-1 stability and assembly.

